# Human Dectin-1- and Dectin-2-targeted DectiSomes are effective against diverse pathogenic fungi

**DOI:** 10.1128/aac.01689-25

**Published:** 2026-03-30

**Authors:** Suresh Ambati, Xiaorong Lin, Zachary A. Lewis, Jesse A. Peter, Richard B. Meagher

**Affiliations:** 1 Department of Genetics, University of Georgia 189619, Athens, Georgia, USA; 2 Department of Microbiology, University of Georgia 189270, Athens, Georgia, USA; University Children's Hospital Münster, Münster, Germany

**Keywords:** human dectins, mucormycosis, DectiSomes, aspergillosis, candidiasis, cryptococcosis, antifungal therapy, liposomes

## Abstract

Annually, aspergillosis, candidiasis, cryptococcosis, and mucormycosis result in approximately 1,500,000, 650,000, 120,000, and 59,000 deaths, respectively. Mortality rates among patients receiving antifungal drug treatment range from 30% to 90%. Therefore, there is an urgent need to improve the efficacy of antifungal drug therapies against infections by these high-priority fungal diseases. *Aspergillus fumigatus*, *Candida albicans*, *Cryptococcus neoformans*, and *Rhizopus delemar* are the most common causative pathogens. We have previously developed DectiSomes, which are liposomes loaded with antifungal drugs and coated with the carbohydrate recognition domains of mouse Dectin-1 and/or Dectin-2. We demonstrated that the murine DectiSomes efficiently bound and killed these pathogens growing *in vitro* and/or in mouse disease models. With the plan to move DectiSomes into the clinic with the human Dectin orthologs, we were concerned that the significant sequence divergence between mouse and human Dectin-1 and Dectin-2 carbohydrate recognition domains could have altered pathogen specificity. Herein, we compared the functionality of the human and mouse Dectin-1 and Dectin-2 orthologs in targeting DectiSomes to these pathogens. Binding and growth inhibition data on *A. fumigatus* and *C. neoformans* supported their functional similarity, while results with *C. albicans* and *R. delemar* indicated some functional divergence. Despite these differences, our results demonstrate that both human and mouse DectiSomes are effective at binding and killing all four diverse fungal pathogens.

## INTRODUCTION

The annual cost from treating fungal infections in the United States exceeds $6 billion, with aspergillosis, candidiasis, cryptococcosis, and mucormycosis being four of the five most common and deadly fungal diseases (1). Globally, these four diseases result in approximately 1,500,000, 650,000, 120,000, and 59,000 deaths annually (2). Drug-treated mortality rates among infected patients range from 30% to 90%. The number of cases of mucormycosis and resulting deaths in India increased by at least an order of magnitude during the recent COVID-19 pandemic (3, 4). *Aspergillus fumigatus*, *Candida albicans*, *Cryptococcus neoformans*, and *Rhizopus delemar* (a.k.a., *Rhizopus arrhizus* or *Rhizopus oryzae*) are the most common causative pathogens, respectively. The dismal statistics among these diseases led the World Health Organization to list these fungi among priority fungal pathogens that needed more research, better diagnostics, and more effective therapeutics (5), which further motivated our research on DectiSomes.

**Fig 1:**
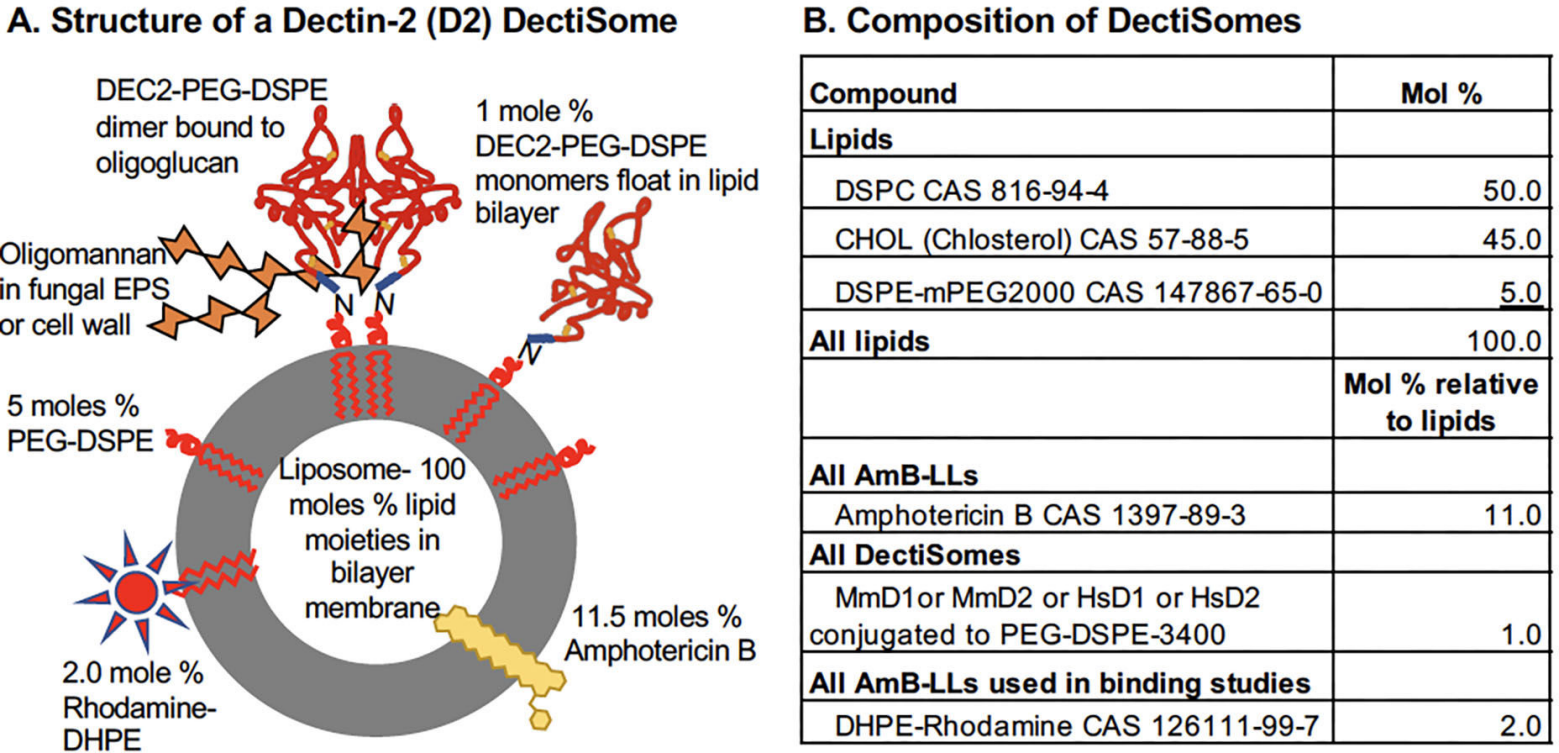
Structure and composition of an anti-fungal DectiSome. (**A**) Structure of a Dectin-2-targeted Amphotericin B (AmB) loaded liposome (D2-AmB-LL, a.k.a., a DectiSome). AmB was loaded remotely in-house to preformed pegylated liposomes to a final concentration of 11.5 mol %. Dectin CRDs were conjugated to PEG-DSPE and inserted via the DSPE lipid moiety at 1 mol %. Rhodamine B-DHPE was inserted via the DHPE lipid moiety at 2 mol % for microscopic detection. Control liposomes, AmB-LLs, lack Dectin. (**B**) The chemical compositions of various preparations DectiSomes employed are presented with mol % values normalized to 100 mol % for the liposomal lipids.

We developed DectiSomes as a new class of antifungal drug loaded liposomes (LLs) targeted to pathogenic fungi by host pathogen receptors (6–8). Dectin-1 (D1) and Dectin-2 (D2) are C-type lectin host pathogen receptors (CLRs), a subclass of pattern recognition receptors, which bind highly diverse oligoglucan and oligomannan structures, respectively. These two CLRs are expressed by several myeloid cell types, and their glycan recognition is an essential part of the innate immune response to pathogenic fungi (9–13). Most fungal pathogens have variously arranged layers of oligoglucans and oligomannans along with other oligoglycans and glycolipids as an integral part of their cell walls and exopolysaccharide (EPS) matrices (14–16). In some cases, the oligoglucans and oligomannans are linked to secreted glycoproteins. These glycan structures make D1 and D2 ideally suited to target antifungal liposomes to the surface of fungal pathogens. We employed the carbohydrate recognition domains (CRDs) of these two Dectins to target DectiSomes to fungal cells, as is shown in a model of a Dectin-2 DectiSome binding to oligomannan on the surface of a fungal pathogen in Fig. 1. We have shown previously that DectiSomes coated with mouse D1 (MmD1) and/or mouse D2 (MmD2) and loaded with the antifungal drug amphotericin B (AmB), MmD1-AmB-LLs, and/or MmD2-AmB-LLs bind and kill *A. fumigatus*, *C. albicans*, *C. neoformans*, and *R. delemar* growing *in vitro* (7, 17–23). These two classes of DectiSomes were also effective at controlling aspergillosis, candidiasis, and cryptococcosis in murine models of disease (24–26). MmD2-AmB-LLs of a smaller diameter and more malleable lipid composition efficiently penetrated the blood-brain barrier to control cryptococcal meningitis (26). The rationale for the efficacy of DectiSomes is the same as that for immunoliposomes used to treat cancer cells. By targeting antifungal drugs in close proximity to pathogenic fungal cells and away from host cells, their efficacy is increased, and treatment regimens can be reduced to safer levels (6, 21).

Our previous studies on DectiSomes employed the carbohydrate recognition domains (CRDs) of *Mus musculus*, MmD1 and MmD2, as targeting polypeptides, because of the necessity to test these liposomes in murine models of fungal infections. In the hope of clinical application of the DectiSome anti-fungal technology, we needed to evaluate the potential of human Dectin-1 (HsD1) and Dectin-2 (HsD2) CRDs as DectiSome targeting polypeptides. Because the amino acid sequences of the CRDs of the mouse and human Dectin-1 and Dectin-2 protein orthologs are only 60% and 72% identical (Fig. S1), the high levels of divergence raised questions about the binding specificity of the human proteins for various fungal pathogens and, hence, their potential efficacy for targeting DectiSomes. Therefore, in this manuscript, we compared the binding and growth inhibitory/killing activities of the AmB-loaded, mouse-targeted DectiSomes MmD1-AmB-LLs and MmD2-AmB-LLs to their human DectiSome counterparts, HsD1-AmB-LL and HsD2-AmB-LLs, against *A. fumigatus*, *C. albicans*, *C. neoformans*, and *R. delemar*. We found that the human DectiSomes were nearly equivalent to the mouse DectiSomes when used against *A. fumigatus* and *C. albicans*, but there were some notable differences against *C. neoformans* and *R. delemar*.

## RESULTS

We cloned, expressed, and affinity purified the CRD regions of human Dectins HsD2 and HsD2 polypeptides (Fig. S1 and S2). We loaded 11.5 mol % Amphotericin B into pegylated liposomes to make untargeted control liposomes, AmB-LLs. AmB-LLs differ from the commercial drug AmBisome primarily in their surface pegylation. The AmB-LLs were loaded with 1 mol % mouse or human Dectin CRDs and with 2 mol % Rhodamine B to make four types of DectiSomes, MmD1-AmB-LLs, MmD2-AmB-LLs, HsD1-AmB-LLs, and HsD2-AmB-LLs. The compositions of the various liposomes employed are summarized in Fig. 1B. To address concerns about the conservation or divergence of cognate ligand specificity of the mouse and human CRD orthologs experimentally, we compared the binding and growth inhibitory/killing activities of the mouse-targeted DectiSomes to their human DectiSome counterparts against *A. fumigatus*, *C. albicans*, *C. neoformans*, and *R. delemar*.

### A. fumigatus

**Fig 2:**
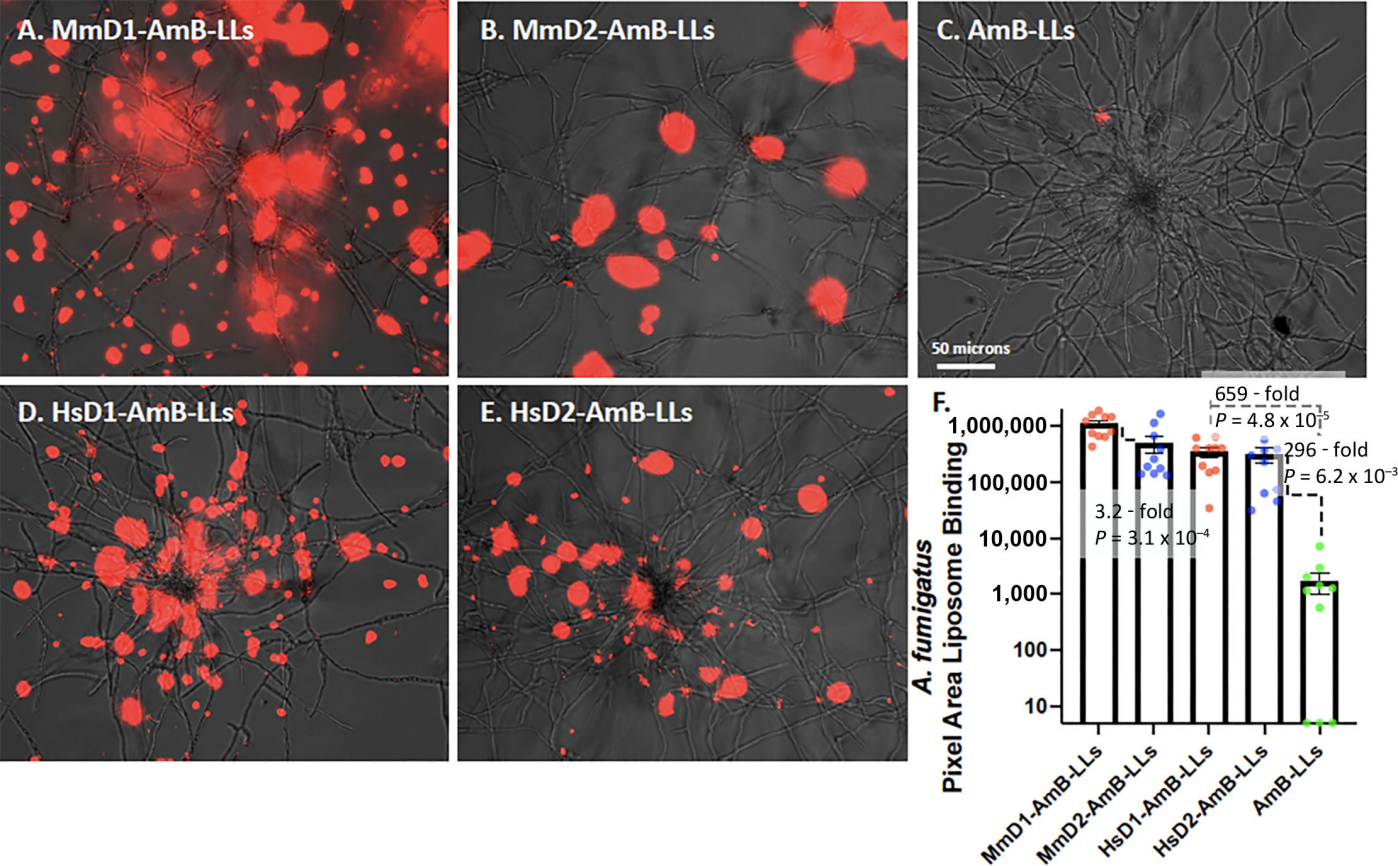
Binding of MmD1-AmB-LLs, MmD2-AmB-LLs, HsD1-AmB-LLs, HsD2-AmB-LLs, and AmB-LLs to *A. fumigatus* hyphae. Images of different liposomes binding to *A. fumigatus*. (**A**) MmD1-AmB-LLs. (**B**) MmD2-AmB-LLs. (**C**) AmB-LLs. (**D**) HsD1-AmB-LLs. (**E**) HsD2-AmB-LLs. Rhodamine red fluorescent liposomes were bound to fixed hyphae. Dectin proteins were delivered at 1 µg/100 µL of buffer (1:100 wt/vol). Cells were photographed at 20× magnification using combined red fluorescence and weak bright-field white light to outline hyphae. A size bar indicates the degree of magnification. (**F**) Quantification of the area of liposome binding. TxRed fluorescent jpeg images (*N* = 10) were quantified using CellProfiler area pipe, and data are presented in a scatter bar plot. The fold change in estimated areas of red fluorescence between critical experimental samples and control AmB-LLs is indicated along with *P* values. Error bars indicate the standard error of the mean.

Fixed hyphal colonies of *A. fumigatus* were treated with fluorescent rhodamine-tagged DectiSomes with the Dectin polypeptides at 1 µg protein:100 µL of buffer (1:100 wt/vol) and with untargeted AmB-LLs diluted to the same degree. AmB-LLs did not bind significantly to hyphal colonies (Fig. 2C and F). By contrast, MmD1-AmB-LLs, MmD2-AmB-LLs, HsD1-AmB-LLs, and HsD2-AmB-LLs all bound in large patches on the surface of colonies (Fig. 2A, B, D and E). Both mouse and human DectiSomes appeared to bind to the exopolysaccharide (EPS) matrix (a.k.a., extracellular matrix) secreted by hyphae and were not particularly localized to the hyphal cell walls. Quantification of the area of fluorescent liposome binding showed that the four classes of DectiSomes bound more than two orders of magnitude more efficiently than AmB-LLs (Fig. 2F). Images and quantification suggested that MmD1-AmB-LL binding was slightly superior to that of the other DectiSomes.

**Fig 3:**
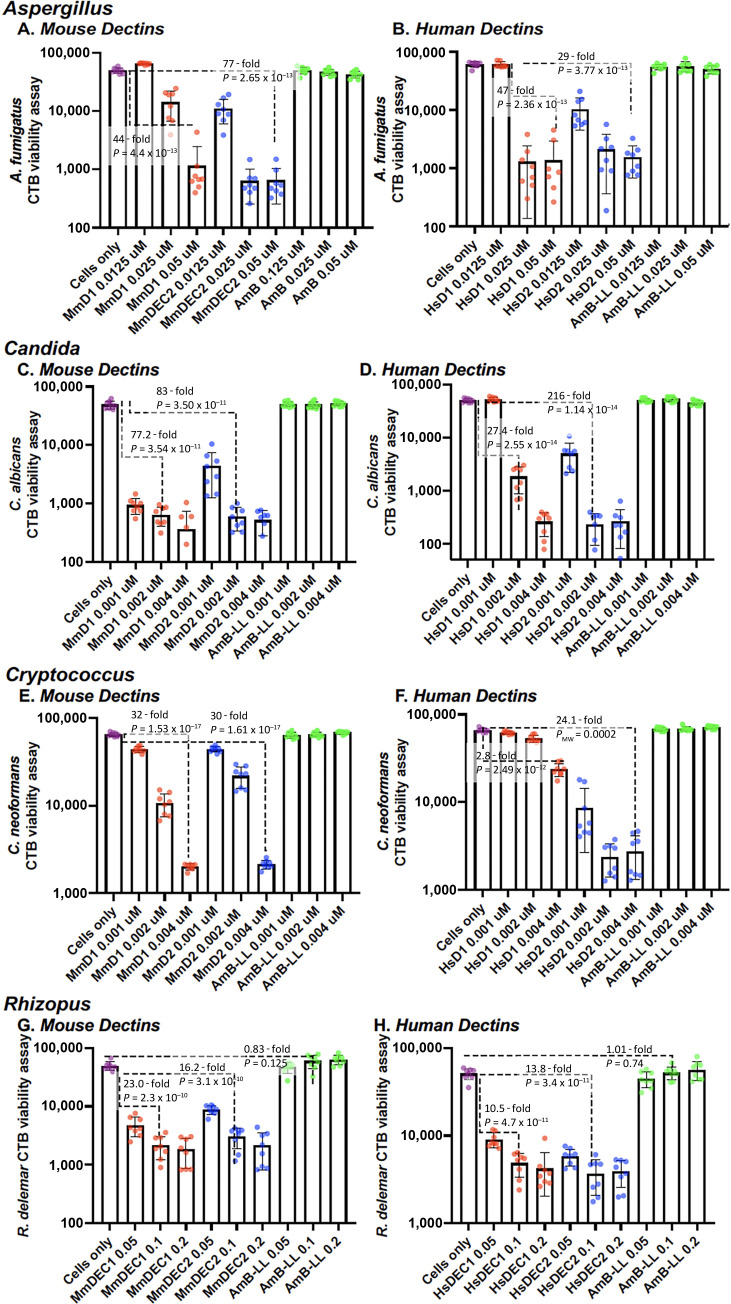
Viability of fungal pathogens after treatment with MmD1-AmB-LLs, MmD2-AmB-LLs, HsD1-AmB-LLs, HsD2-AmB-LLs, and AmB-LLs. Residual cell viability was measured with the CTB reagent after treatment with various DectiSomes and AmB-LLs delivering a range of AmB concentrations. (**A and B**) *A. fumigatus* was treated with various mouse and human DectiSomes delivering 0.0125, 0.025, and 0.05 µM AmB. (**C and D**) *C. albicans* was treated with various mouse and human DectiSomes delivering 0.001, 0.002, and 0.004 µM AmB. (**E and F**) *C. neoformans* was treated with various mouse and human DectiSomes delivering 0.001, 0.002, and 0.004 µM AmB. (**G and H**) *R. delemar* was treated with various mouse and human DectiSomes delivering 0.05, 0.1, and 0.2 µM AmB. The fold changes in average CTB fluorescence values between example comparisons are indicated in scatter bar plots along with *P* or *P*_MW_ values. Error bars indicate the standard error of the mean. *N* = 8 samples for each bar.

The growth inhibitory and killing activities of the mouse and human DectiSomes to *A. fumigatus* were compared by treating growing cultures overnight with liposomal reagents delivering three different concentrations of AmB, 0.0125, 0.025, and 0.05 µM (Fig. 3). Cell viability was assayed with CellTiter-Blue (CTB) reagent, which measures electrochemical redox activity in live cells with an intact electron transport system. In concordance with their lack of binding, AmB-LLs were ineffective at inhibiting growth at all three concentrations of AmB tested. All four types of DectiSomes, MmD1-AmB-LLs, MmD2-AmB-LLs, HsD1-AmB-LLs, and HsD2-AmB-LLs, inhibited growth at 0.025 and 0.05 µM AmB (Fig. 3A and B). Both the mouse and human Dectin-2-targeted reagents were most effective, showing significant inhibitory activity even at 0.0125 µM. This comparable fungal killing activity among the mouse or human Dectin-targeted DectiSomes might be unexpected given the slightly higher binding activities of the MmD1-AmB-LLs targeted reagent over that of the other DectiSomes. Overall, there were no dramatic differences between the mouse or human Dectin-targeted DectiSomes against *A. fumigatus*. Two replicate experiments gave similar results for the binding and killing experiments.

### C. albicans

**Fig 4:**
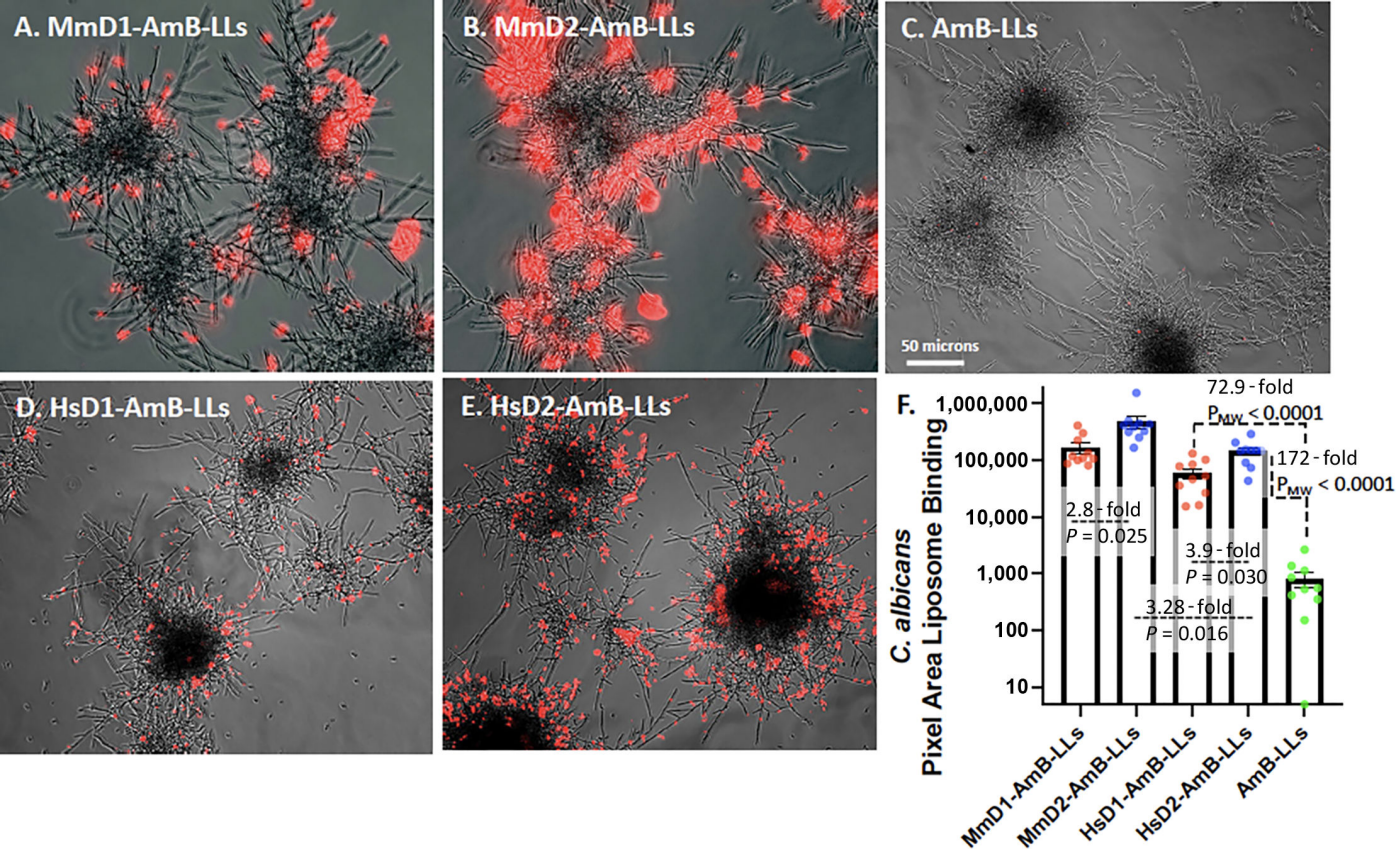
Binding of MmD1-AmB-LLs, MmD2-AmB-LLs, HsD1-AmB-LLs, HsD2-AmB-LLs, and AmB-LLs to *C. albicans* hyphal colonies. Images of different liposome binding to *C. albicans*. (**A**) MmD1-AmB-LLs. (**B**) MmD2-AmB-LLs. (**C**) AmB-LLs. (**D**) HsD1-AmB-LLs. (**E**) HsD2-AmB-LLs. Rhodamine red fluorescent liposomes were bound to fixed hyphae. (**F**) Quantification of the area of liposome binding. See details in the legend to Fig. 2.

AmB-LLs did not bind significantly to fixed hyphal colonies of *C. albicans* (Fig. 4C and F). By contrast, MmD1-AmB-LLs, MmD2-AmB-LLs, HsD1-AmB-LLs, and HsD2-AmB-LLs bound in large patches all over the surface of the hyphal colonies, but there were obvious differences in the observed levels of binding (Fig. 4A, B, D and E). Quantification of the area of fluorescent liposome binding showed that the four classes of DectiSomes bound one to two orders of magnitude more efficiently than AmB-LLs (Fig. 4F). Mouse and human Dectin-2-targeted DectiSomes bound approximately threefold more strongly than their Dectin-1 counterparts, and MmD2-AmB-LLs bound threefold more strongly than HsD2-AmB-LLs (Fig. 4F).

The growth inhibitory activities of the mouse and human DectiSomes to *C. albicans* were compared by treating cells overnight with liposomal reagents delivering 0.001, 0.002, and 0.004 µM AmB to the growth media (Fig. 3C and D). Cell viability was assayed with CTB reagent. In concordance with their lack of binding, AmB-LLs were ineffective at inhibiting growth at all three tested concentrations of AmB. All four types of DectiSomes, MmD1-AmB-LLs, MmD2-AmB-LLs, HsD1-AmB-LLs, and HsD2-AmB-LLs, efficiently inhibited growth when delivering 0.002 and 0.004 µM AmB. Experiments with higher concentrations of AmB concentrations also supported orders of magnitude better killing by DectiSomes than AmB-LLs (Fig. S3). All the DectiSomes, except HsD1-AmB-LLs, were effective even at 0.001 µM AmB. Although the mouse and human D2-targeted DectiSomes showed superior binding relative to HsD1 reagents, this only translated to stronger growth inhibition data for human reagents. Two replicate experiments gave similar results for the binding and killing experiments.

### C. neoformans

**Fig 5:**
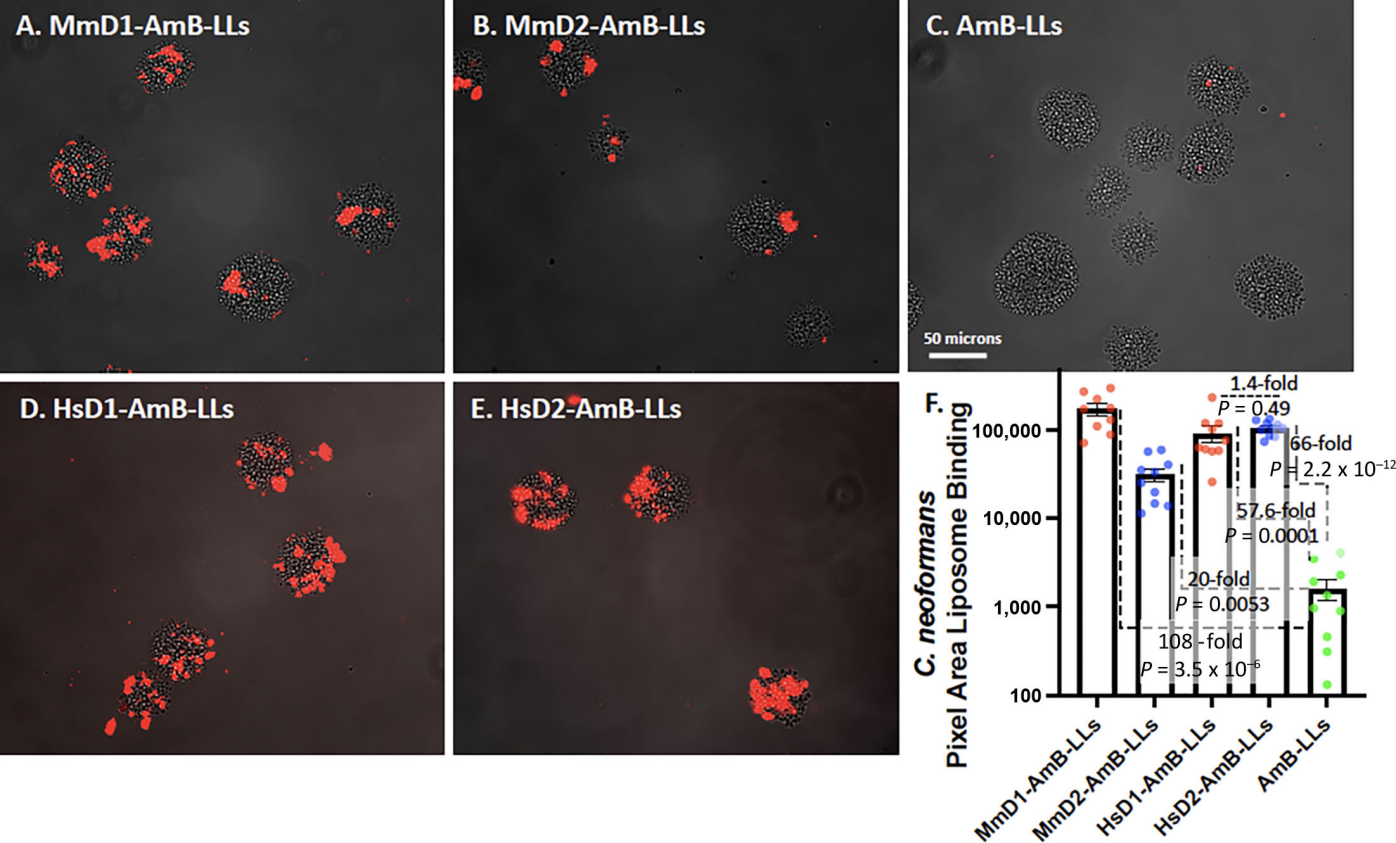
Binding of MmD1-AmB-LLs, MmD2-AmB-LLs, HsD1-AmB-LLs, HsD2-AmB-LLs, and AmB-LLs to *C. neoformans* colonies. Images of different liposome binding to *C. neoformans* colonies. (**A**) MmD1-AmB-LLs. (**B**) MmD2-AmB-LLs. (**C**) AmB-LLs. (**D**) HsD1-AmB-LLs. (**E**) HsD2-AmB-LLs. Rhodamine red fluorescent liposomes were bound to fixed colonies grown on the surface of agar. Images were taken top down through a coverslip at 20× magnification (numerical aperture [N.A.] 0.8 lens). (**F**) Quantification of the area of liposome binding. See details in the legend to Fig. 2.

*C. neoformans* was grown on the surface of agar for binding studies, because the colonies of yeast cells do not efficiently stick to the surface of plastic microtiter plates. Agar plugs were removed and fixed with formalin. AmB-LLs did not bind significantly to fixed colonies (Fig. 5C and F). By contrast, MmD1-AmB-LLs, MmD2-AmB-LLs, HsD1-AmB-LLs, and HsD2-AmB-LLs all bound in small patches within the colonies (Fig. 5A, B, D and E). Quantification of the area of fluorescent liposome binding showed that the four classes of DectiSomes bound one to two orders of magnitude more efficiently than AmB-LLs (Fig. 5F). MmD1-AmB-LLs bound more strongly than HsD1-AmB-LLs, while HsD2-AmB-LLs bound much more effectively than MmD2-AmB-LLs.

The growth inhibitory activities of the mouse and human DectiSomes to *C. neoformans* were compared by treating cells overnight with liposomal reagents delivering 0.001, 0.002, and 0.004 µM AmB to the growth media (Fig. 3E and F). In concordance with their lack of binding, AmB-LLs were ineffective at inhibiting growth at all three concentrations of AmB. MmD1-AmB-LLs were more effective at growth inhibition than HsD1-AmB-LLs, consistent with the slightly higher level of MmD1-AmB-LL binding. HsD1-AmB-LLs were only effective at inhibiting growth at 0.004 µM AmB. By contrast, HsD2-AmB-LLs is more effective than MmD2-AmB-LLs at growth inhibition, consistent with its stronger binding (Fig. 5F). In contrast to their very different binding activities, MmD1-AmB-LLs and MmD2-AmB-LLs exhibited similar levels of dose-dependent growth inhibition. We were surprised that such low concentrations of AmB were required to show concentration-dependent growth inhibition of *C. neoformans*, because the binding data for *C. neoformans* appeared less remarkable than it was for *A. fumigatus* or *C. albicans*. In addition, we had not anticipated significant differences in the activity of the two human reagents based on their comparable binding data. Two replicate experiments gave similar results for the binding and killing experiments.

### R. delemar

**Fig 6:**
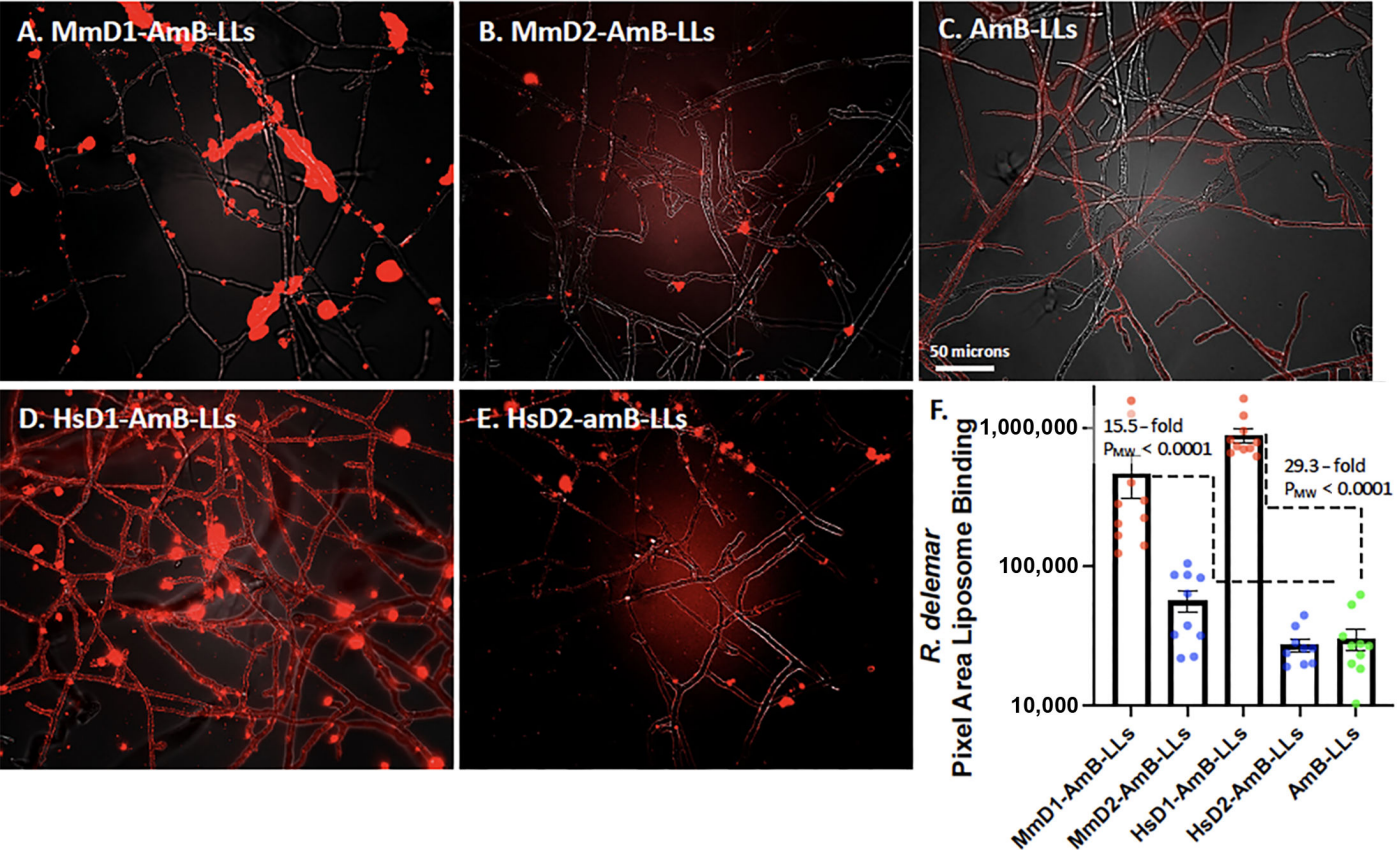
Binding of MmD1-AmB-LLs, MmD2-AmB-LLs, HsD1-AmB-LLs,HsD2-AmB-LLs, and AmB-LLs to *R. delemar* hyphae. Images of different liposome binding to *R. delemar* hyphae. (**A**) MmD1-AmB-LLs. (**B**) MmD2-AmB-LLs. (**C**) AmB-LLs. (**D**) HsD1-AmB-LLs. (**E**) HsD2-AmB-LLs. Rhodamine red fluorescent liposomes were bound to fixed hyphae grown on the surface of agar. Images were taken top down through a coverslip at 20× magnification (N.A. 0.8 lens). (**F**) Quantification of the area of liposome binding. See details in the legend to Fig. 2.

*R. delemar* was grown on the surface of agar, because its cells do not stick to the surface of plastic microtiter plates (19). Agar plugs were removed and fixed with formalin. Binding assays were partially compromised by the fact that some hyphae penetrate the agar surface and are both less available to liposome binding and to photographic detection, which is limited by the microscopic depth of field. AmB-LLs bound very weakly to the surface of some *R. delemar* hyphae (Fig. 6C), although the low level of binding did not appear to contribute measurably to our quantitative binding assays (Fig. 6F). MmD1-AmB-LLs and HsD1-AmB-LLs bound strongly in numerous patches in what appears to be the EPS (Fig. 6A and D), while the level of MmD2-AmB-LLs and HsD2-AmB-LLs binding was insignificant (Fig. 6B, E and F). Interestingly, HsD1-AmB-LLs also bound strongly to the surface of the hyphae themselves (Fig. 6D), while MmD1-AmB-LLs did not, suggesting there is a distinct difference in the binding specificity of the two Dectin-1 orthologs. Binding by both MmD1-AmB-LLs and HsD1-AmB-LLs was order of magnitude more efficient than for AmB-LLs (Fig. 6F). HsD1-AmB-LLs bound slightly more strongly than MmD1-AmB-LLs, which may be accounted for by its binding to hyphae in addition to EPS.

The growth inhibitory activities of the mouse and human DectiSomes to *R. delemar* were compared by treating cells overnight with liposomal reagents delivering 0.05, 0.1, and 0.2 µM AmB to the growth media (Fig. 3G and H). AmB-LLs were ineffective at inhibiting growth at all three tested concentrations of AmB. In contrast to the distinctly different binding activities of the Dectin-1- and Dectin-2-targeted reagents, MmD1-AmB-LLs, MmD2-AmB-LLs, HsD1-AmB-LLs, and HsD2-AmB-LLs were all efficient at inhibiting growth at all three concentrations relative to AmB-LLs and inhibition showed modest dose dependence. Two replicate experiments gave similar results for the binding and killing experiments. Ten- to 100-fold higher AmB concentrations began to resolve dose-dependent differences in AmB-LL killing of all four species, as shown for *R. delemar* in Fig. S4.

### Binding to EPS vs the cell wall

**Fig 7:**
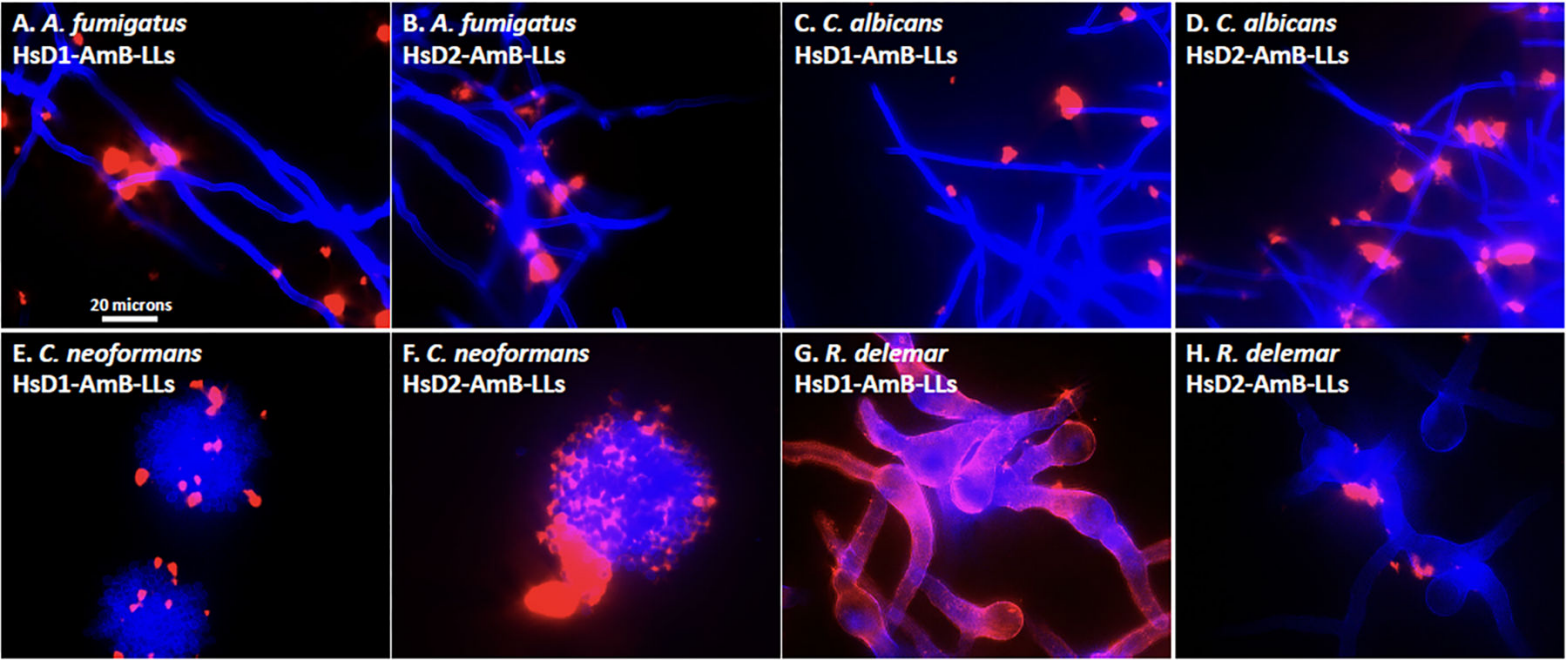
Higher magnification images of all four fungal pathogens treated with HsD1-AmB-LLs and HsD2-AmB-LLs and stained with calcofluor white. *A. fumigatus* (**A and B**), *C. albicans* (**C and D**), *C. neoformans* (**E and F**), and *R. delemar* (**G and H**) were treated with HsD1-AmB-LLs and HsD2-AmB-LLs, respectively. Cells were photographed at 60× magnification (N.A. 1.35 lens) using combined red fluorescence (100% intensity, 500 to 1,000 msec) to identify DectiSomes and blue fluorescence of calcofluor white to highlight the chitin in the cell wall (100% intensity, 60 msec). The blue fluorescence of some images was enhanced in Adobe Photoshop to better define fungal cells. A size bar indicates the degree of magnification.

It was sometimes difficult to discern if DectiSome binding was localized to the cell wall or to secreted EPS in the images taken at 20× magnification (Fig. 2, 4 to 6). This was particularly true for *C. neoformans* and *R. delemar*. Therefore, we took higher magnification 60× images of the human Dectin-targeted reagents, HsD1-AmB-LLs, and HsD2-AmB-LLs, binding to all four species, in which the chitin in the cell wall was stained with calcofluor white to better define cell boundaries (Fig. 7). From these images, it appeared that the binding to *A. fumigatus*, *C. albicans*, and *C. neoformans* was indeed to the EPS and not to their cell walls. By contrast, Fig. 7G suggests that most of HsD1-AmB-LL binding to *R. delemar* was to the cell wall itself or immediately adjacent to it, along with binding to small patches of EPS. Multiple 60× images of the human DectiSome staining of these four species showed consistently similar results.

## DISCUSSION

### Divergence of the mouse and human Dectin orthologs

Mice and humans diverged from common ancestry approximately 80 million years ago (27), so some divergence in the cognate ligand specificities of their Dectin orthologs is expected. Indeed, the CRDs of the mouse and human Dectin-1 and Dectin-2 protein orthologs are 40% and 28% different in their amino acid sequences (Fig. S1). This level of divergence necessitated an experimental validation of the efficacy of the human orthologs in guiding antifungal DectiSomes to diverse fungal pathogens for their use in the clinic for the following reasons. First, this level of divergence means that if the mouse Dectin polypeptides are used in humans, they will likely cause host immune response, resulting in the equivalent of the often toxic Human Anti-Mouse Antibody (HAMA) response as documented in the early development of mouse monoclonal antibody therapeutics (28). Typically, a protein with more than 20% sequence divergence from the host ortholog is highly immunogenic (29). Hence, we felt that clinical studies on DectiSomes should be done with human Dectins not mouse Dectins. Second, because there are numerous other pathogen receptors besides these two Dectins and some also recognize oligoglucans and oligomannans (30), we were concerned that the cognate ligand specificity of the mouse and human Dectins for the glycan structures presented by particular fungal pathogens may have been lost, sub-functionalized, and perhaps completely replaced by other pathogen receptors in the intervening 80 million years, since the orthologs had a common ancestry. Our results suggest that most of their functions have been conserved and that the Dectins from both species are moderately comparable at enhancing the killing of fungal pathogens by DectiSomes.

### Glycan targets of Dectin-1 and Dectin-2 in various pathogens

Recall that Dectin-1 binds glucans with a β-(1-3) and β-(1-6) linked side chains, while Dectin-2 binds diverse α-mannans including galactomannans (31). Although all four fungal pathogens express oligoglucans and oligomannans, they differ dramatically in their relative percentages to each other and their relative percentages to other glycans, glycolipids, and glycoproteins that compose the cell wall and EPS (14–16). The four fungal species may also differ in the order of stacking of glycan layers in their cell envelope such that an outer layer might block liposome access to an inner layer of glycans. We will discuss these concerns separately for each pathogen.

#### A. fumigatus

The cell wall and EPS of *A. fumigatus* hyphae have complex inner layers rich in β-glucans and outer layers containing galactomannan among other polysaccharides (14, 32–34). The EPS is composed of approximately 40% protein, 43% polysaccharide, 3% aromatic compounds such as melanin, and up to 14% lipid (35). Extracellular proteins are most likely glycoproteins and may contain ligands of the Dectins. Our binding experiments do not distinguish between binding to glycoproteins and binding to oligoglycans. The EPS of *A. fumigatus* is essential to pathogenicity and to its adherence to solid and semisolid surfaces (36, 37). The EPS is not detected when grown in liquid cultures (38). Mouse and human Dectin-1- and Dectin-2-targeted DectiSomes bound efficiently to and were effective at inhibiting the growth of *A. fumigatus* hyphae (Fig. 2 and 3A and B), supporting the potential application of human Dectins in the clinic against *A. fumigatus*

#### C. albicans

Although 50% of the *C. albicans’* cell wall is composed of oligoglucans, an oligomannan matrix lies outside of the oligoglucan layer (14). The oligomannan layer is reported to effectively mask oligoglucans from recognition by the host innate and adaptive immune systems (39–41), including binding by the oligoglucan-specific C-type lectin Dectin-1 and by oligoglucan-specific antibodies (40, 42). Our data on DectiSome binding to fixed hypha are moderately consistent with this layering in that both mouse MmD2-AmB-LLs and human HsD2-AmB-LLs bound significantly better than their Dectin-1-targeted counterparts (Fig. 4F). Unexpectedly, both mouse and human D1- and D2-targeted DectiSomes were similarly and highly efficient at killing *C. albicans* by at least two orders of magnitude relative to AmB-LLs, even when delivering very low sub-micromolar concentrations of AmB. Replicate experiments with still lower concentrations of AmB did not resolve any significant differences among the Dectins. Despite their 100 nm diameter spherical dimensions, the Dectin-1-targeted DectiSomes must have penetrated through the oligomannan matrix to the oligoglucan layer of live cells to efficiently deliver AmB.

#### C. neoformans

It is difficult to define the composition of the *C. neoformans* cell wall and EPS, both because they are highly variable under different growth conditions and because it is challenging to define where the cell wall glycans leave off and the capsule begins. The cell wall of *C. neoformans* contains primarily alpha (1–3) glucans, which are essential to virulence in mice (43). The capsule of *C. neoformans*, which includes the proximal components of a highly extended EPS, may comprise 80% to 90% of the cell volume and can extend more than 10 microns from the cell wall (44, 45). The capsule is composed primarily of two complex polysaccharides, glucuronoxylomannan (GXM) and galactoxylomannan (GalXM), and a small proportion of mannoproteins (46, 47). It was reported that Dectin-2 does not recognize GXM or GalXM, but Dectin-2 does appear to recognize some mannan component of *C. neoformans* (48). We showed previously that MsD2-AmB-LLs bound strongly to components within the GXM matrix and that MsD2-AmB-LLs were efficient at killing cryptococcal cells *in vitro* (22). In this context, our head-to-head comparison of the four DectiSomes produced some interesting results. First, MmD1-AmB-LLs bound far more efficiently to *C. neoformans* colonies than MmD2-AmB-LLs (Fig. 5), but they were indistinguishable in their efficiency of killing, when delivering nanomolar concentrations of AmB (Fig. 3E and F). Second, this strong difference in binding was not observed between HsD1-AmB-LLs and HsD2-AmB-LLs (Fig. 5F). Third, HsD2-AmB-LLs were the most efficient at reducing cell viability. And, by contrast, HsD1-AmB-LLs were by far the least active. Despite these differences, MmD1-AmB-LLs, MmD2-AmB-LLs, and HsD2-AmB-LLs were orders of magnitude more effective at killing cryptococcal cells compared to untargeted AmB-LLs. And, it was surprising that in spite of the predominance of oligoglucans reported in the cell wall of *C. neoformans* (43), Dectin-1-targeted reagents appeared to bind to the EPS in preference to the cell wall (Fig. 5A, D and 7E).

#### R. delemar

*R. delemar* belongs to the ancient and highly diverse Mucorales order of fungi. We were only able to infer indirectly the cell wall and EPS composition from the limited published data that are available. Polysaccharides in the hyphal cell walls of Mucorales including *R. delemar* are composed primarily of glucose units and to a much lesser extent mannose units (49–51). However, the EPS matrices of some *Mucor* spp. have more balanced ratios of glucose and mannose units (51, 52). Both mouse and human Dectin-1 DectiSomes bound far more strongly to fixed *R. delemar* hyphae than Dectin-2 reagents, consistent with their specificity for oligoglucans and the high glucose content of the EPS. It was surprising that HsD1-AmB-LLs bound so efficiently to the cell walls of *R. delemar*, while MmD1-AmB-LLs did not (Fig. 6A, D and 7G). By contrast, growth inhibition studies showed that mouse MmD1-AmB-LLs, MmD2-AmB-LLs, HsD1-AmB-LLs, and HsD2-AmB-LLs all had strong inhibitory activity.

It is only logical to assume that a targeted antifungal liposome can’t improve killing unless there is increased binding to target cells, which will increase local drug concentrations. There are a few reasons that data for the Dectin specificity of binding and inhibition/killing by *R. delemar* and *C. albicans* might not align. Cells are only exposed to reagent liposomes for 1 h during the binding assays. But during killing and inhibition assays, live cells are exposed overnight, giving the liposomes a longer time to penetrate through layers of polysaccharide. In addition, fungal cell walls such as those of *C. albicans* are highly dynamic, constantly being remodeled, which can periodically mask and/or unmask various cell wall components such as oligoglucans and oligomannans, the cognate ligands of Dectin-1 and Dectin-2, (14, 15, 53). During the live cell assays, we added the respective liposomal reagent when the cells/spores were at the germling stage or starting to germinate and throughout the growth cycle to mature hyphae or colonies, whereas the binding studies were performed on fixed cells at one stage of development. Thus, differential remodeling and differential expression of glycans in different developmental stages may increase or decrease DectiSome binding and killing.

### Conclusion

Both mouse and human Dectin-1- and Dectin-2-targeted DectiSomes were an order of magnitude more effective at killing all four fungal pathogens than AmB-LLs. Considering that the mouse and human Dectin orthologs were highly divergent in their primary sequences, it is not surprising that we detected some minor differences in their binding affinities and inhibitory activities of these reagents to different fungal species. The most notable qualitative difference among all our binding data were the apparent affinity of HsD1-AmB-LLs for the cell walls of *R. delemar*, which was not shared with MmD1-AmB-LLs. However, our data strongly suggest that despite such differences, the CRDs of mouse and human Dectin-1 and Dectin-2 orthologs can effectively guide liposomes to diverse fungal pathogens and kill them. Furthermore, DectiSomes targeted by human Dectins have the potential to kill fungal pathogens in the clinic.

## MATERIALS AND METHODS

### Fungal species, strains, and media

*A. fumigatus CEA10* (54, 55) was grown in Vogel’s minimal medium (56) + 1% glucose + 100 µg/mL kanamycin and streptomycin (22). *C. albicans* SC5314 (57) and *C. neoformans* H99-alpha (58) were grown in RPMI with no indicator dye (Sigma-Millipore R8755) + antibiotics (22). *R. delemar* 99-880 (ATCC MYA-4621) was grown in liquid media or on agar plates in RPMI + 0.165 M MOPS (3-(N-morpholino) propane sulfonic acid) (Sigma-Aldrich, Cat# M1254, St. Louis, MO, USA) adjusted to pH 7.0 with NaOH + antibiotics (19, 59). Spores or cells were diluted to approximately 50,000/mL in media and grown at 37°C. Plastic 24- and 96-well microtiter plates received 1,000 and 90 µL of the cell suspensions, respectively, with the exception that 200 µL of *C. neoformans* and *R. delemar* was spread on 150 mm diameter agar plates.

### Preparation of human Dectin

The CRDs of human Dectins HsD1 (Dectin-1 isoform A, NP_922938.1) and HsD2 (Dectin-2 isoform 2, NP_001304928.1) polypeptides (Fig. S1C and D) were cloned, expressed, and purified (Fig. S2) as described for their mouse orthologs (22, 23). The average preparation yield of HsD1 and HsD2 per liter Luria broth grown cells was 7 mg and 28 mg, respectively. The two human isoforms we employed are the two closest homologs of the mouse proteins. The human and mouse polypeptides were coupled to the lipid carrier DSPE-PEG-3400-NHS (Nanosoft Polymers, 1544-3400) via the NHS reactive moiety, following the protocols used previously for the mouse polypeptides (22, 23).

### Constructing DectiSomes

Pegylated liposomes were purchased from FormuMax Sci. Inc. (F20203). AmB-LLs, HsD1-AmB-LLs, and HsD2-AmB-LLs were made in-house and prepared as described previously for MmD1-AmB-LLs and MmD2-AmB-LLs, respectively (22, 23) (Fig. 1B). Plain AmB-LLs with no Dectins were used as controls. Some liposomes were also loaded with 2 mol % Rhodamine B as in the model of a Dectin-2-targeted DectiSome in Fig. 1. Rhodamine-tagged versions were used for binding studies. All types of liposomes prepared in-house were stored for no more than 3 months at 4°C before use.

### Assays of liposome binding

Cells were grown until colonies of *A. fumigatus* and *C. albicans* in 24-well plates reached 50 to 500 microns in diameter, washed once in Dulbecco’s phosphate buffered saline (DPBS) (Corning 21-031-CV, DPBS), fixed for 30 min with 4% formalin (J.T. Baker, #2106-01) freshly diluted into DPBS, washed thrice 5 min each with DPBS, blocked for 1 h with modified liposomal binding buffer (LDB2) (23) supplemented with 5% bovine serum albumin (BSA) + 1 mM BME, stained for 1 h with DectiSomes in modified LDB2 and Dectin polypeptides at 1 µg/100 µL, washed again thrice with binding buffer, and photographed. Untargeted control AmB-LLs were diluted to the same liposome concentration. When cells were treated with calcofluor white (Bayer Corp., Blankophor BBH, CAS 4193-55-9, Pittsburgh, PA, USA), it was used in 1:1,000 dilution from a 25 mM calcofluor white (CW) stock, just before the last wash. Colonies of *C. neoformans* and *R. delemar* were grown on agar plates. Seven mm diameter agar plugs were loosened with a sterile cork-borer, transferred to 24-well microtiter plates, and treated similarly to the other fungi except the washes and treatments were extended to 1 h each to remove formalin (19). Microscopy used to measure liposome binding was performed on an ECHO Revolve microscope (model #RVSF1000/Revolve R4) examining cells bottom up in 24-well microtiter plates (Greiner Bio-One Cellstar, Cat#662160) using a 20× N.A. 0.45 lens or in a special thin F-bottom 96-well microplate (Greiner Bio-One Cat#655090) using a 60× N.A. 1.35 lens or top down through a coverslip for cells grown on agar using a 20× N.A. 0.8 lens or the 60× lens. Images of liposome staining were captured in the Texas red (TxRed) channel plus the visible light channel using the bright-field phase condenser. In some cases, cells were also stained with calcofluor white and images recorded in the DAPI blue fluorescence channel. For some images, the DAPI blue calcofluor white channel was enhanced to better reveal cell boundaries. Merged images were recorded as Revolve’s zoverlay.jpg files. The area of fluorescent liposome staining was quantified from TxRed channel jpeg images using the Cell Profiler (v4.2.8)-based pipeline, AreaPipe (v. 5), which we developed specifically for this purpose (7). Quantitative binding data were presented as the pixel area of fluorescent staining. Binding assays were performed on fixed cells to ensure that cells stayed bound to their growth substrates during the procedure.

### Assays of cell viability

Spores or cells (4,500/mL) were plated at 90 µL per well in 96-well microtiter plates. DectiSomes or AmB-LLs were diluted into growth media at 10 times the desired final µM concentrations of AmB. Ten microliters of reagent was added to the 90 µL of cells once the cells started to germinate. Sixteen to 20 h later, 20 µL of CTB reagent (resazurin, Promega Cat#G8081) was added. Resorufin, the product of the REDOX reaction, was quantified at Ex485/Em590 in a fluorescent microtiter plate reader (BioTek Synergy HT). Plates were incubated with reagent at 37°C for 2 to 3 h until the control untreated cell samples reached maximum amount of fluorescence that could be measured, approximately 70,000 to 90,000 fluorescent units (FLUs). The average fluorescence from wells with media and CTB reagent, but lacking cells, was subtracted as background. Initial screening was performed with AmB concentrations of 0.05 to 2 µM, and then the dose was reduced until we observed some degree of dose dependence among the various DectiSome treatments. In preliminary assays, the liposomes were washed out after 2-h exposure with the assumption that it would better resolve differences between bound DectiSomes and unbound AmB-LLs as done in our previous publications on the mouse reagents used against *A. fumigatus*, *C. albicans*, and/or *C. neoformans* (22, 23). *R. delemar* sticks to plastic pipette tips, and most of the cells are removed by washing steps (19). In fact, washing caused a wide scatter in the resulting viability data for all four pathogens and only modestly improved measured differences for any pathogen. In all assays presented herein, the liposomes were not washed out.

### Computation and preparation of figures

The initial data output for pixel area of liposome staining or FLU for viability assays was managed using Microsoft Excel (v. 16.100.1). Most of the data were normally distributed; hence, the Student’s two-tailed *t*-test was used to estimate *P* values (T.TEST in Excel) (60). GraphPad Prism 9 (v. 9.5.0) was used to prepare scatter bar plots and estimate standard errors from the mean. In cases where the data for at least one sample in a comparison appeared to be non-parametric in their distribution, *P* values were estimated using the Mann–Whitney test(61) in Prism 9 and were indicated as *P*_MW_ values. Prism sets the limit of *P*_MW_ values at <0.0001. Figures were prepared in Microsoft Power Point (v. 16.100.2), exported as pdf files, captured from the pdfs, and then assembled and resized as JPEG files in Adobe Photoshop (v. 25.12.3) and saved as TIFF images for publication.

## Data Availability

All new data discussed are included within this article, and all information and data from outside sources are appropriately cited.

## SUPPLEMENTAL MATERIAL

Supplemental material aac.01689-25-s0001.pdf Fig. S1 to S4.

ASM does not own the copyrights to Supplemental Material that may be linked to, or accessed through, an article. The authors have granted ASM a non-exclusive, world-wide license to publish the Supplemental Material files. Please contact the corresponding author directly for reuse.
