# Supplementary material for: Human Dectin-1- and Dectin-2-targeted DectiSomes are effective against diverse pathogenic fungi
Source: Antimicrob Agents Chemother. 2026 Mar 30;70(5):e01689-25. doi: 10.1128/aac.01689-25 (PMC13148054; doi:10.1128/aac.01689-25)

Supplemental Fig. SF1. The sequences of mouse and human Dectin-1 and Dectin-2 employed herein and sequence comparisons of their carbohydrate recognition domains (CRDs)

S1A. MmD1 blast HsD1 CRDs

Best hit was C-type lectin domain family 7 member A isoform A [Homo sapiens]
Sequence ID: NP_922938.1 Length: 247Number of Matches: 1

See 8 more title(s) See all Identical Proteins(IPG)

Related Information
Gene-associated gene details
AlphaFold Structure-3D structure displays
Genome Data Viewer-aligned genomic context
Identical Proteins-Identical proteins to NP_922938.1
Range 1: 69 to 246GenPeptGraphics
Next Match
Previous Match
Alignment statistics for match #1

Score	Expect	Method	Identities	Positives	Gaps
215 bits(548)	4e-70	Compositional matrix	106/178(60%)	127/178(71%)	1/178(0%)

```
MmD1  1    FWRHNSGRNPEEKDSFLSRNKENH-KPTESSLDEKVAPSKASQTTGGFSQSCLPNWIMHG  59
            WR NSG N  E   FLSRNKENH +PT+SSL++ V P+KA +TTG  S  C PNWI++
HsD1  69   IWRSNSGSNTLENGYFLSRNKENHSQPTQSSLEDSVTPTKAVKTTGVLSSPCPPNWIIYE  128

MmD1  60   KSCYLFSFSGNSWYGSKRHCSQLGAHLLKIDNSKEFEFIESQTSSHRINAFWIGLSRNQS  119
           KSCYLFS S NSW GSKR C QLG++LLKID+S E  FI  Q SS   N+FWIGLSR Q+
HsD1  129  KSCYLFSMSLNSWDGSKRQCWQLGSNLLKIDSSNELGFIVKQVSSQPDNSFWIGLSRPQT  188

MmD1  120  EGPWFWEDGSAFFPNSFQVRNAVPQESLLHNCVWIHGSEVYNQICNTSSYSICEKELA  177
           E PW WEDGS F  N FQ+R    QE+    NCVWIH S +Y+Q+C+  SYSICEK+ +
HsD1  189  EVPWLWEDGSTFSSNLFQIRTTATQENPSPNCVWIHVSVIYDQLCSVPSYSICEKKFS  246
```

S1B. MmD2 blast HsD2 CRDs

MmDEC2, MmD2 blast human proteins. Best hit was the C-type lectin domain family 6 member A isoform 2 [Homo sapiens]
Sequence ID: NP_001304928.1 Length: 179Number of Matches: 1
Related Information
Gene-associated gene details
AlphaFold Structure-3D structure displays
Genome Data Viewer-aligned genomic context
Range 1: 19 to 179GenPeptGraphics Next Match Previous Match
Alignment statistics for match #1

Score	Expect	Method	Identities	Positives	Gaps
250 bits(639)	4e-85	Compositional matrix.	117/163(72%)	131/163(80%)	2/163(1%)

```
MmD2  4    QPSRRLYELHTYHSSLTCFSEGTMVSEKMWGCCPNHWKSFGSSCYLISTKENFWSTSEQN  63
           +  +RL ELH+YHSSLTCFSEGT V    WGCCP  WKSFGSSCY IS++E  WS SEQN
HsD2  19   ETGKRLSELHSYHSSLTCFSEGTKV--PAWGCCPASWKSFGSSCYFISSEEKVWSKSEQN  76
```

```
MmD2  64   CVQMGAHLVVINTEAEQNFITQQLNESLSYFLGLSDPQGNGKWQWIDDTPFSQNVRFWHP  123
           CV+MGAHLVV NTEAEQNFI QQLNES SYFLGLSDPQGN  WQWID TP+ +NVRFWH
HsD2  77   CVEMGAHLVVFNTEAEQNFIVQQLNESFSYFLGLSDPQGNNNWQWIDKTPYEKNVRFWHL  136

MmD2  124  HEPNLPEERCVSIVYWNPSKWGWNDVFCDSKHNSICEMKKIYL  166
            EPN   E+C SIV+W P+ WGWNDV C+++ NSICEM KIYL
HsD2  137  GEPNHSAEQCASIVFWKPTGWGWNDVICETRRNSICEMNKIYL  179
```

S1C. MmD1 The modified CRD and stalk region of murine (Mus musculus) Dectin-1 amino acid sequence employed in DectiSomes and derived from NCBI. The CRD and stalk region is highlighted in yellow. 199 a.a., 22.4 kDa, pI 7.74.
MAHHHHHHVGTGSGKGKGSGSGFWRHNSGRNPEEKDSFLSRNKENHKPTESSLDEKVAPSKASQTTGGFSQSCLPNWIMHG
KSCYLFSFSGNSWYGSKRHCSQLGAHLLKIDNSKEFEFIESQTSSHRINAFWIGLSRNQSEGPWFWEDGSAFFPNSFQVRN
AVPQESLLHNCVWIHGSEVYNQICNTSSYSICEKELA

S1D. MmD2 The modified CRD and stalk region of murine (Mus musculus) Dectin-2 amino acid sequence employed in DectiSomes derived from NCBI. The CRD and stalk region is highlighted in yellow. 189 a.a., 21.7 kDa, pI 6.33.
MAHHHHHHVGTGSGKGKGSGSGIMDQPSRRLYELHTYHSSLTCFSEGTMVSEKMWGCCPNHWKSFGSSCYLISTKENFWST
SEQNCVQMGAHLVVINTEAEQNFITQQLNESLSYFLGLSDPQGNGKWQWIDDTPFSQNVRFWHPHEPNLPEERCVSIVYWN
PSKWGWNDVFCDSKHNSICEMKKIYLA

S1E. HsD1 The modified CRD and stalk region of human(Homo sapiens) Dectin-1 amino acid sequence employed in DectiSomes derived from NCBI. The CRD and stalk region is highlighted in yellow. 202 a.a., 22.5 kDa, pI 7.67
MAHHHHHHYGTGSGKGKGSGSGIWRSNSGSNTLENGYFLSRNKENHSQPTQSSLEDSVTPTKAVKTTGVLSSPCPPNWIIY
EKSCYLFSMSLNSWDGSKRQCWQLGSNLLKIDSSNELGFIVKQVSSQPDNSFWIGLSRPQTEVPWLWEDGSTFSSNLFQIR
TTATQENPSPNCVWIHVSVIYDQLCSVPSYSICEKKFSMA

S1F. HsD2 The modified CRD and stalk region of human(Homo sapiens) Dectin-2 amino acid sequence employed in DectiSomes derived from NCBI. The CRD and stalk region is highlighted in yellow. 187 a.a., 21.1 kDa, pI 6.40.
MAHHHHHHVGTGSGKGKGSGSGTYGETGKRLSELHSYHSSLTCFSEGTKVPAWGCCPASWKSFGSSCYFISSEEKVWSKSE
QNCVEMGAHLVVFNTEAEQNFIVQQLNESFSYFLGLSDPQGNNNWQWIDKTPYEKNVRFWHLGEPNHSAEQCASIVFWKPT
GWGWNDVICETRRNSICEMNKIYLA

Supplemental Fig. SF2. SDS PAGE analysis of different stages in the affinity purification of HsD1 and HsD2 and density scan of 5 ug lanes.

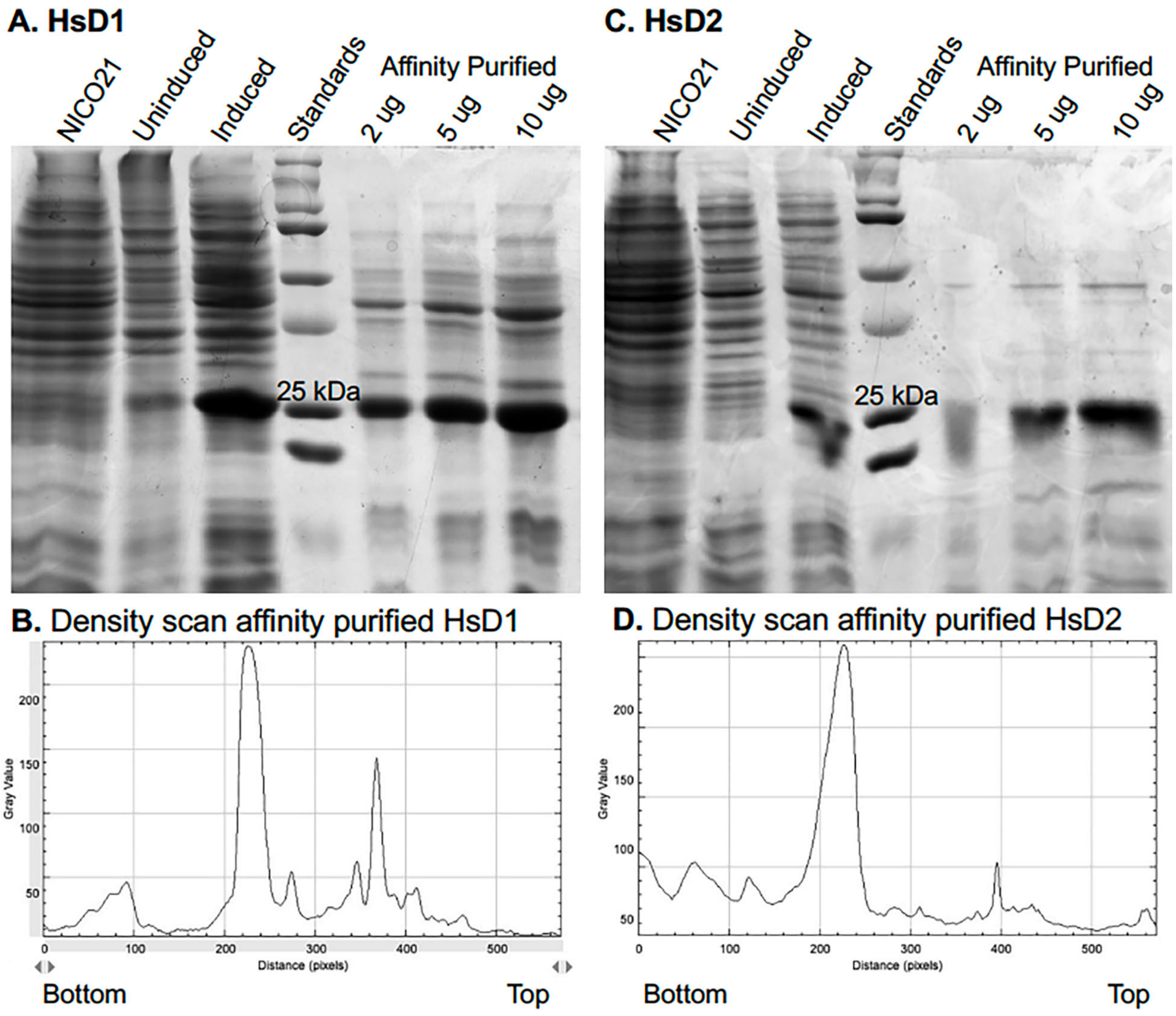

Supplemental Fig. SF3. Viability of *C. albicans* after treatment with MmD1-AmB-LLs, MmD2-AmB-LLs, HsD1-AmB-LLs, HsD2-AmB-LLs, and AmB-LLs delivering slightly higher concentrations of AmB than shown in Fig. 3C and 3D.

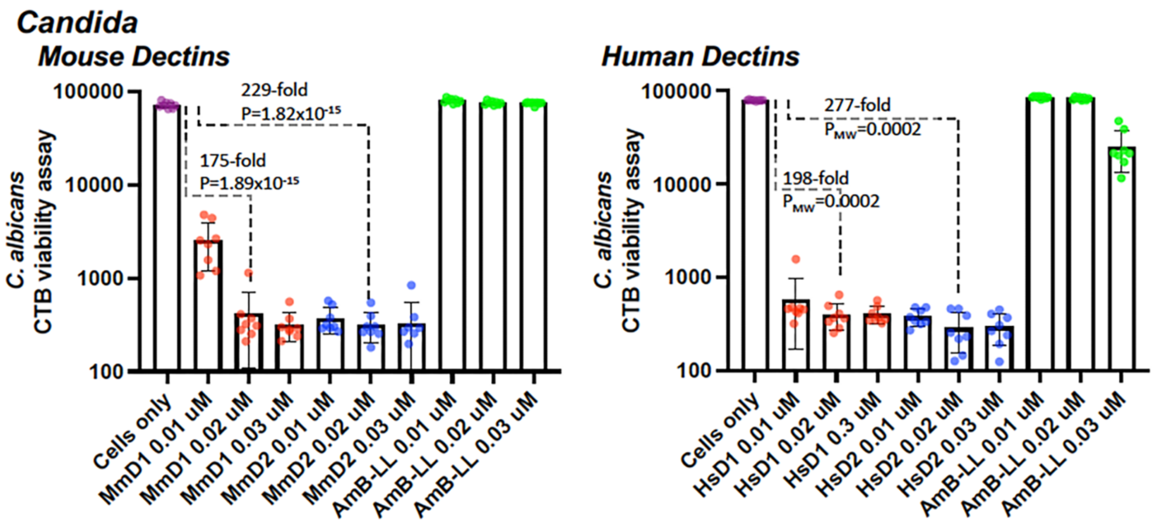

Supplemental Fig. SF4. Viability of *R. delemar* after treatment with MmD1-AmB-LLs, MmD2-AmB-LLs, HsD1-AmB-LLs, HsD2-AmB-LLs, and AmB-LLs delivering slightly higher concentrations of AmB than shown in Fig. 3G and 3H.

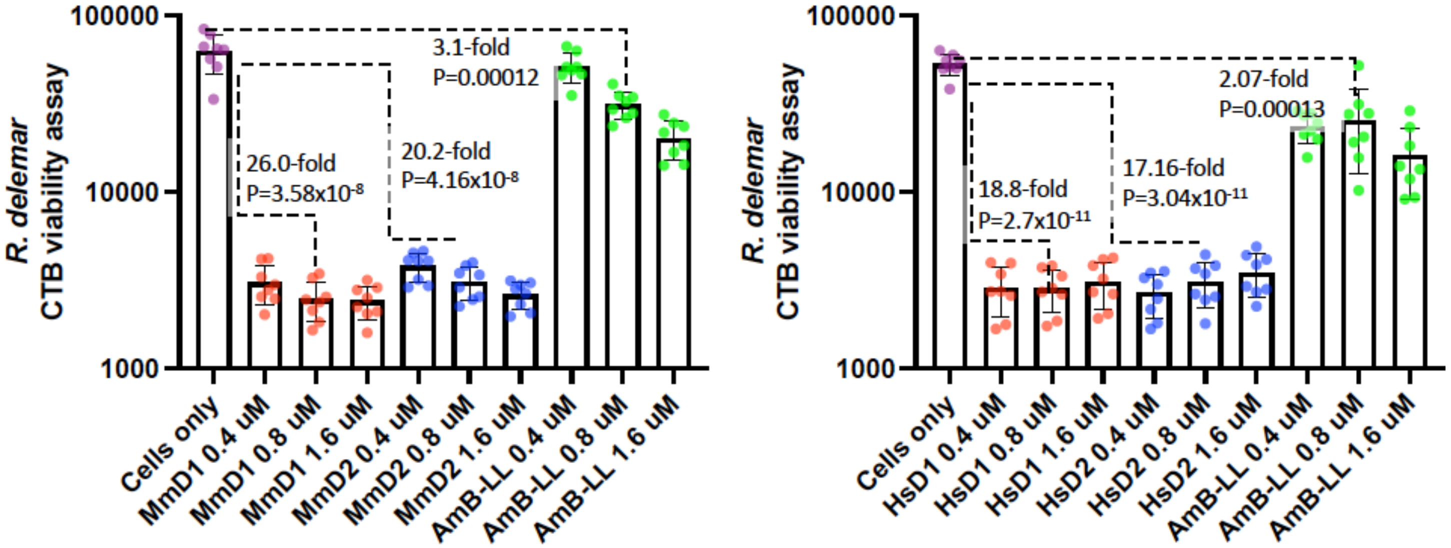

Supplement: Supplemental material — Fig. S1 to S4. [file aac.01689-25-s0001.pdf]
